# Transferring Knowledge from MM to QM: A Graph Neural Network-Based Implicit Solvent Model for Small Organic Molecules

**DOI:** 10.1021/acs.jctc.5c00728

**Published:** 2025-07-28

**Authors:** Paul Katzberger, Felix Pultar, Sereina Riniker

**Affiliations:** Department of Chemistry and Applied Biosciences, 27219 ETH Zürich, Vladimir-Prelog-Weg 2, Zürich 8093, Switzerland

## Abstract

The conformational ensemble of a molecule is strongly influenced by the surrounding environment. Correctly modeling the effect of any given environment is, hence, of pivotal importance in computational studies. Machine learning (ML) has been shown to be able to model these interactions probabilistically, with successful applications demonstrated for classical molecular dynamics. While first instances of ML implicit solvents for quantum-mechanical (QM) calculations exist, the high computational cost of QM reference calculations hinders the development of a generally applicable ML implicit solvent model for QM calculations. Here, we present a novel way of developing such a general machine-learned QM implicit solvent model by transferring knowledge obtained from classical interactions to QM, emulating a QM/MM setup with electrostatic embedding and a nonpolarizable MM solvent. This has the profound advantages that neither QM/MM reference calculations nor experimental data are required for training and that the obtained graph neural network (GNN)-based implicit solvent model (termed QM-GNNIS) is compatible with any functional and basis set. QM-GNNIS is currently applicable to small organic molecules and describes 39 different organic solvents. The performance of QM-GNNIS is validated on NMR and IR experiments, demonstrating that the approach can reproduce experimentally observed trends unattainable by state-of-the-art implicit-solvent models paired with static QM calculations.

## Graphical abstract

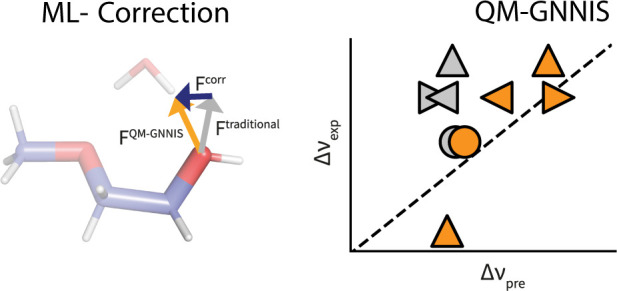

## Introduction

The physical and chemical properties of molecules are the result of their three-dimensional (3D) structure. Importantly, flexible molecules do not have a single 3D structure but rather adopt a Boltzmann-weighted ensemble of different conformations.^1^ Therefore, computational approaches in disciplines like spectroscopy,^2–4^ medicinal chemistry,^5,6^ and stereoselective synthesis^7^ need to take the conformational flexibility into account to provide accurate predictions. This, in turn, means that the accurate but fast prediction of conformational ensembles has long been appreciated as one of the prime objectives and challenges of computational chemistry.

The conformational ensemble of a molecule can be obtained from its free-energy landscape. Given an accurate Hamiltonian and within the limit of infinite sampling, molecular dynamics (MD)^8–10^ is an established method to generate free-energy landscapes. However, for all but the smallest molecules, this method requires extensive sampling to reach convergence, even when more modern enhanced sampling methods are used. *In silico* conformer generators^11–14^ forego the limitations posed by sampling methods and use geometric relationships between atoms to swiftly enumerate 3D-structure estimates from the topological graph. When conformer generators are paired with a method to estimate the free energy of the resulting conformers, a Boltzmann-weighted ensemble of molecular structures is obtained.

In the condensed phase, the calculation of molecular ensembles is complicated by the presence of not only intramolecular interactions but also interactions with the surrounding solvent. Small differences in the nature of the solvents may lead to substantially different ensembles. Approaches that include explicit solvent molecules in the calculation are considered most rigorous to describe interactions of solute and solvent. However, the large number of explicit molecules comes with a steep increase in computational cost, especially for calculations that involve more expensive quantum-mechanical (QM) Hamiltonians, which scale poorly with increasing system size. Furthermore, explicit-solvent models were shown to have a lower sampling efficiency due to the additional degrees of freedom introduced by the solvent, which requires extensive sampling, and also the increase in viscosity.^15^

Multiresolution approaches like QM/MM^16–18^ or, more recently, ML/MM^19–23^ aim to maintain a high level of accuracy for the description of the solute while solvent molecules are approximated with classical force fields. These approaches are often fruitful when an explicit description of the electronic structure of the solute is desired, for example to study chemical reactions. However, these methods show the same limitations as classical MD simulations and often require vast computational resources.

Implicit solvent models aim to represent the effect a solvent exerts on a solute by an electrostatic continuum. This approximation not only reduces the computational burden due to a much smaller number of explicit particles in the system but also significantly accelerates sampling efficiency due to the instantaneous averaging of solvent configurations and reduced viscosity.^15,24^ Many implicit solvent models, especially those that are used in conjunction with QM methods, consider the solute’s electron density when solving the Poisson–Boltzmann equation.^25,26^ Examples include the conductor-like screening (COSMO),^27^ the conductor-like polarizable continuum (CPCM),^28^ and the solvation model based on density (SMD)^29^ models. A more complex solvent model that aims to include explicit-solvent effects was coined COSMO-RS.^30,31^ Starting from perfectly screened molecules in the conductor-like approach, the COSMO-RS model uses methods from statistical thermodynamics to pair charged surface segments. Due to the relatively high computational burden associated with solving the Poisson–Boltzmann equation, force-field methods are typically paired with implicit solvent models that are based on the generalized Born (GB) model, which does not consider the solute’s electron density but rather atomic monopoles. Examples of these models are GB-HCT,^32^ GB-OBC,^33^ GB-Neck,^34^ and GB-Neck2.^35^ Examples of GB models employed in QM calculations include SM8 ^36^ and SM12.^37^

In previous studies, we have developed a graph neural network (GNN) based implicit solvent model (GNNIS), which was trained on forces extracted from classical MD simulations with explicit solvent.^38–40^ While GNNIS yielded excellent results compared to the reference explicit-solvent simulations as well as experimental observables, replicating this approach directly for QM calculations is made prohibitively expensive by the necessary reference QM/MM (or *ab initio* MD) calculations for the training data. While transfer learning (i.e., taking a ML model trained on one task as a starting point for another) may allow for a reduction in training data, acquiring a diverse training set would still require a substantial computational effort. In addition, a specific functional and basis set would need to be chosen, potentially limiting the approach’s applicability to these specific choices. For this reason, we aimed to develop an approach that extracts knowledge from GNNIS in classical simulations, which we found to reproduce solute–solvent interactions well,^38,39^ and transfers it to QM calculations as a correction with respect to traditional implicit solvation. This procedure has the crucial benefit that no further training data is required, and the approach should be applicable to any functional and basis set combination.

For this approach, we rely on the similarity between classical and QM-based implicit solvent models. In both cases, the solvent is modeled as a dielectric continuum interacting with the solute. While the way this continuum interacts with a given solute is modeled differently, deviations from a more accurate explicit-solvent description mostly stem from the lack of correct modeling of the solute–solvent interface. While at a larger distance, modeling a solvent as a continuum is a good approximation, the binary nature of actual solvent molecules is no longer accurately described at short distances. We denote this as the explicit-solvent effect, which we hypothesize to be of similar magnitude for classical and QM/MM (with a nonpolarizable MM solvent) simulations. When studying the behavior of classical MD simulations, we realized that it is mainly this effect that leads to the largest deviations between a traditional implicit solvent and the explicit-solvent ground truth.^38–40^ For classical force fields, we could already show that the explicit-solvent effect is well reproduced by the GNNIS model, which was trained on a large set of reference forces of ∼370,000 molecules in 39 organic solvents.^40^

In order to assign a free-energy contribution to the explicit-solvent effect, ΔΔ*G* ^corr^, we define it as the difference between the true solvation free energy of a compound in solution and the solvation free energy estimated based on a continuum model that neglects the explicit-solvent effect. Taking the classical GNNIS model as a good representation of the true solvation free energy of a classically described molecule and the GB-Neck2 model as a continuum-based estimate, ΔΔ*G* ^corr^ can then be approximated for the identically described molecule as,

1 $$\Delta \Delta G^{\mathrm{corr}}=\Delta G^{\mathrm{GNNIS}}-\Delta G^{\mathrm{GB‐Neck2}}$$

where Δ*G* ^GNNIS^ is the free-energy contribution calculated with the classical GNNIS model and Δ*G* ^GB‑Neck2^ is the free-energy contribution calculated with the traditional implicit solvent model GB-Neck2.^35^ Under the assumption that this difference is approximately equal for MM solutes and for QM solutes, we combined it with the QM-based CPCM^28^ solvent to develop a machine-learned QM implicit solvent, which we denote QM-GNNIS. We note that a formal connection between GB models like GB-Neck2 and apparent surface-charge models like CPCM exists,^41^ which motivates the combination of these two models. As a separability of the two contributions is assumed, the benefit of the proposed approach lies in the direct accessibility of energies, gradients, and Hessians necessary to optimize structures and calculate relevant experimental properties. A schematic representation of the approach is shown in Figure 1. The force contribution of the solvent on the hydroxy oxygen of 2-methoxy-ethanol is indicated. This compound is known to feature a strong explicit-solvent effect with water, as water can form a pseudo seven-membered ring conformation with the molecule. In these instances, our hypothesis is that the correction force *F* ^corr^ leads to more meaningful solute–solvent interactions compared to continuum-based methods on their own.

**Figure 1:**
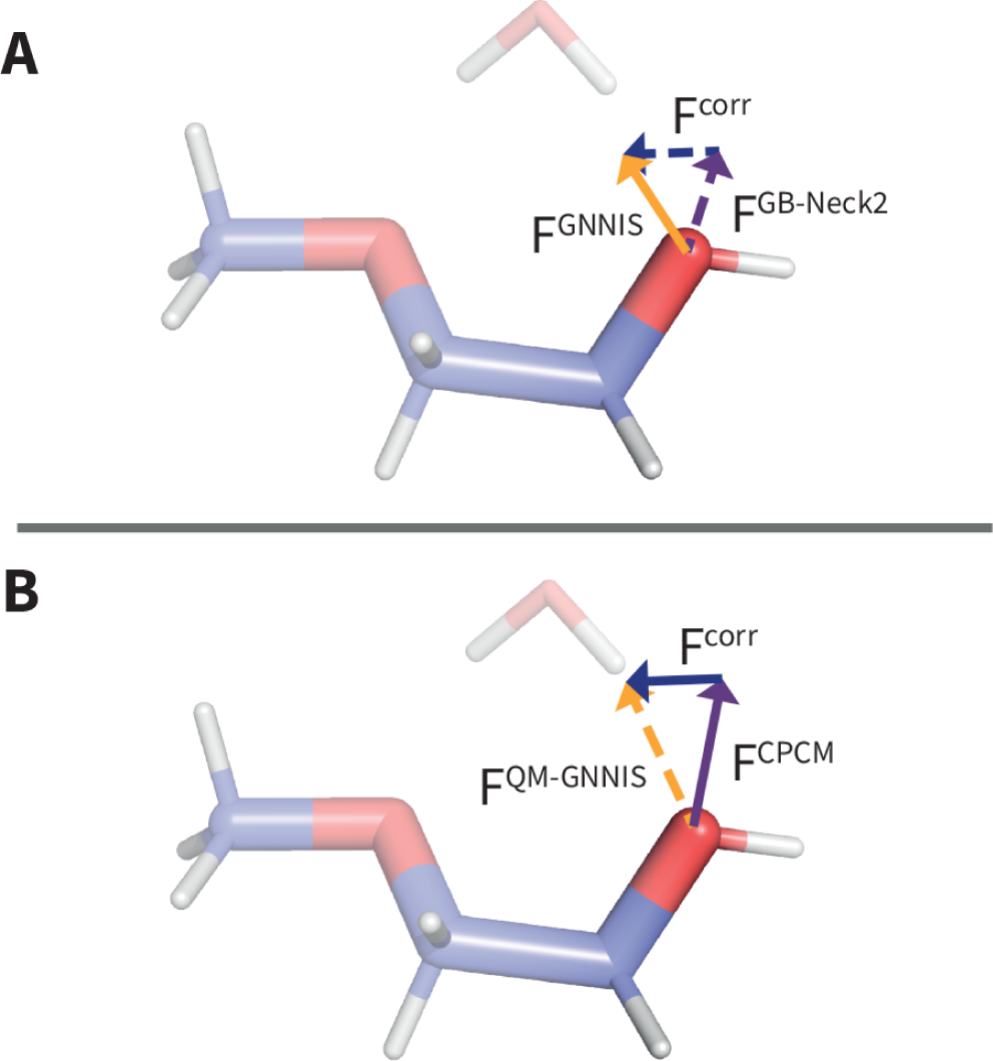
Schematic representation of the proposed approach. Theoretical force vectors acting on the hydroxy oxygen are indicated. (A) Forces predicted based on a classical force field. The force of the classical GNNIS model is indicated by the solid orange vector. The forces of the traditional GB-Neck2 implicit solvent based on the same force field, as well as the difference to the GNNIS force vector (i.e., the correction term), are indicated in dashed purple and blue arrows, respectively. (B) Forces predicted for the QM-based implicit solvent model. The force of the QM-based CPCM implicit solvent model is shown by the solid purple arrow. The classical correction force is indicated by the solid blue arrow. The resulting QM-GNNIS force (i.e., the sum of the two aforementioned contributions) is represented by the dashed orange arrow. Note that the correction term *F* ^corr^ is identical for panels A and B.

We emphasize that the proposed QM-GNNIS approach does not constitute an implicit solvent model that targets the accuracy of *ab initio* QM calculations, but rather the approach emulates QM/MM simulations with electrostatic embedding and a nonpolarizable MM solvent. Note that in our setting, the solute is only polarized based on a continuum approach (i.e., the CPCM solvent), with *F* ^corr^ added to the gradients of the QM atoms. Including explicit-solvent effects in this manner is not exhaustive but provides an improvement over traditional implicit solvent models routinely used in QM calculations.

## Results and Discussion

ML models are typically evaluated based on their ability to reproduce reference calculations and are then, if applicable, compared against experimental results. While this validation strategy has been used successfully in many recent studies,^42–47^ including our own,^38–40^ the comparison of QM-GNNIS against the computational ground truth (i.e., explicit-solvent *ab initio* MD or QM/MM MD) is too expensive for the system sizes, simulation lengths, and level of theory we are using. For this reason, we rely on experimental data to evaluate the performance of the approach. To rigorously study the proposed methodology, a large number of ∼200 experimental measurements of 24 different test systems are studied. As a point of reference, QM calculations with the state-of-the-art implicit solvent models SMD^29^ and openCOSMO-RS^48^ are compared to the proposed approach. Note that we chose SMD solvent (and not CPCM) for the comparison as it was fitted to better reproduce solvation free energies of solutes in different solvents and can thus be thought of as an improvement over CPCM.^29^ This fitting is achieved by using solvent-specific parameters that should better describe solvent characteristics (e.g., hydrogen bonding) not captured by the dielectric permittivity of the solvent. As this procedure may already capture some of the explicit solvent effects, we anticipated that the combination of SMD and the correction from QM-GNNIS may overestimate some interactions. Therefore, we decided to combine QM-GNNIS with the simpler CPCM model. To ensure that this is indeed the case, we have performed reference calculations for the set of molecular balances that support this hypothesis (see Supporting Information Section S1).

### Conformational Preference of Molecular Balances

To understand how the different implicit-solvent models behave, a set of 22 molecular balances for which experimental free-energy differences between conformers of the central amide group have been determined in ref 49 were studied using the BP86 functional^50^ and the def2-SVP basis set^51^ with D4 correction^52^ (Figure 2A). First, classically optimized structures were taken as a starting point for QM optimizations with the SMD implicit-solvent model. The free energies of the optimized structures were evaluated with SMD and openCOSMO-RS. They also served as starting structures for the evaluation with QM-GNNIS, which was used to refine the conformers further before the free energies were estimated. The comparison between the predicted and experimentally observed free-energy differences for molecular balances A1, B1, C1, and D1 is shown in Figure 2B, and the results for all molecular balances are provided in Figures S2–S4.

**Figure 2:**
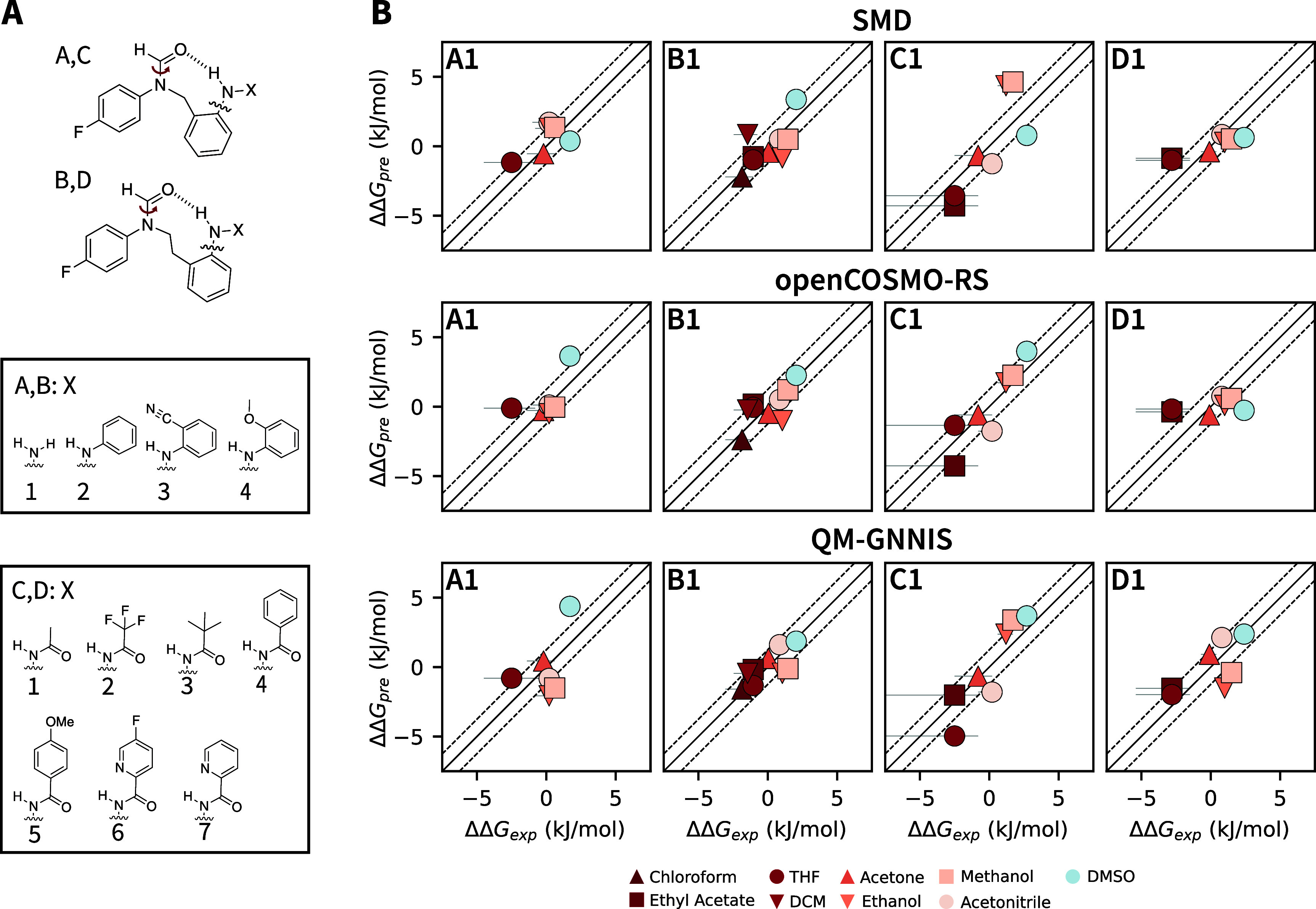
(A) Schematic representation of the molecular balances.^49^ The studied rotation of the amide group is indicated in red. (B) Comparison of the predicted versus observed free-energy differences, ΔΔ*G*, for the QM calculations with SMD (top), openCOSMO-RS (middle), and QM-GNNIS (bottom). The experimental values were taken from ref 49. The solid and dashed black lines indicate identity and deviations of half *k*_b_T, respectively. The color indicates the dielectric permittivity of the solvent (red: apolar, blue: polar).

Overall, all implicit-solvent models provide decent agreement between the predicted ΔΔ*G* and the experimental values. To compare the models quantitatively, we calculated the Pearson correlation coefficient (PCC) for all balance/solvent combinations (top panel of Figure 3). While the SMD and QM-GNNIS models show a similar correlation, the openCOSMO-RS model performs significantly worse.

**Figure 3:**
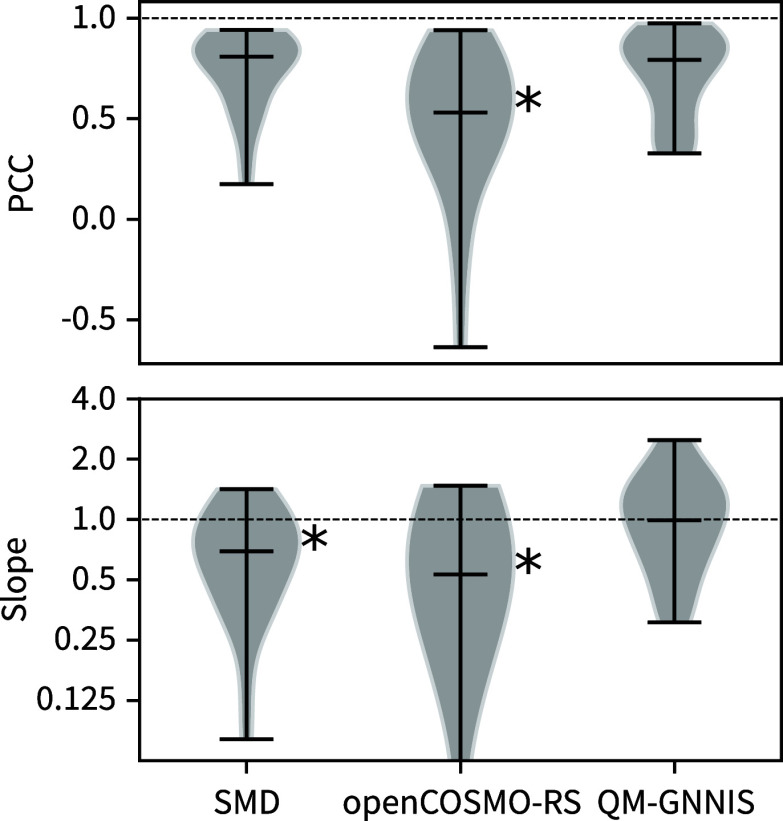
(Top) Pearson correlation coefficient (PCC) between the experimentally observed ΔΔ*G* values and the predictions based on QM calculations with SMD, openCOSMO-RS, and QM-GNNIS. The black star indicates a significant (*p* < 0.05) difference with respect to the other models. (Bottom) Slopes between the experimentally observed ΔΔ*G* values and the predictions based on QM calculations with SMD, openCOSMO-RS, and QM-GNNIS. Black stars indicate a significant (*p* < 0.05) difference between the distributions and the perfect slope of 1.

While the correlation between the experimental and predicted ΔΔ*G* values is of significance, the strength of the solvent interaction is of equal importance. Molecular balance D1, for instance, shows an excellent correlation with PCC = 0.92 for the SMD solvent model, even though the differences between the solvents are too small, leading to a slope of 0.34. Therefore, we also evaluated the capability of an implicit-solvent model to reproduce the correct relative interaction strength by calculating the slope between experiment and prediction (bottom panel of Figure 3). This analysis revealed that while the SMD model achieves good correlations, it systematically underestimates the difference in Δ*G* between the different solvents, manifesting in slopes that are significantly below one. While the same trend was observed for the openCOSMO-RS model, the QM-GNNIS model captures the differences well, resulting in slopes centered around one.

In this context, it is noteworthy that predictions obtained with a classical force field and the GNNIS model also show excellent agreement with experiment as demonstrated in our previous study.^40^ However, the largest outlier in this study was molecular balance A3, which features a nitrile group in close proximity to a key hydrogen bond interacting with the ketone. Interactions involving higher multipoles that may be relevant in this case are not captured by classical fixed-charge force fields,^54^ potentially leading to the large deviations. Using QM calculations with QM-GNNIS, in contrast, reproduced the experimental trends much better (Figure 4), highlighting the benefits of QM-based methods for the description of more complex molecular interactions.

**Figure 4:**
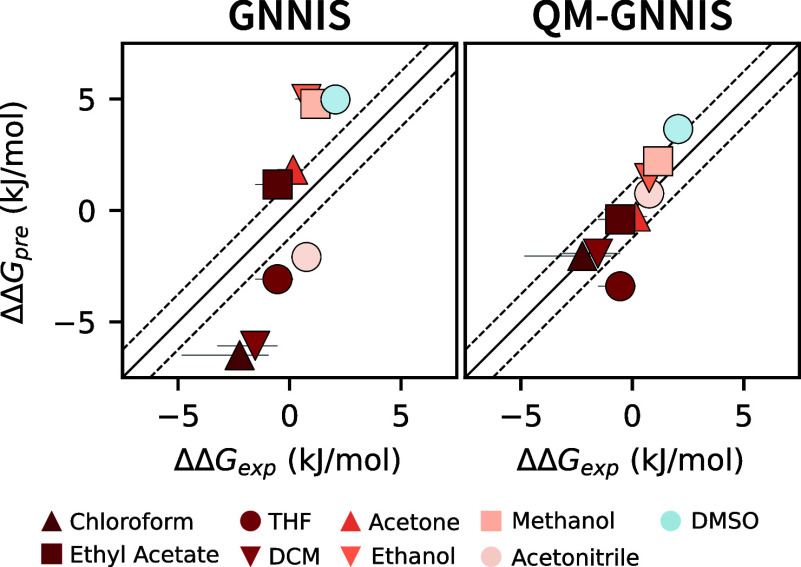
Comparison of predicted versus observed free-energy differences, ΔΔ*G*, for the classical approach (OpenFF^53^ with GNNIS) and for QM calculations with QM-GNNIS for molecular balance A3. The experimental values were taken from ref  ^49^ classical data from ref 40. The solid and dashed black lines indicate identity and deviations of half *k*_b_T, respectively. The color indicates the dielectric permittivity of the solvent (red: apolar, blue: polar).

### Solvent Effects in NMR and IR Spectroscopy

In case of the molecular balances above, the experimentally observed trend correlates with the dielectric permittivity of the solvent, i.e., apolar solvents such as chloroform, ethyl acetate, THF, and DCM showed a stronger preference for the intramolecular hydrogen bond while polar solvents such as methanol, ethanol, and DMSO showed a stronger preference for the “open” state. To further investigate the behavior of the different implicit-solvent models, we focused on examples where more complex solute–solvent interactions are at play.

#### NMR Measurements

The conformational preference of the two small molecules 2-methoxy-ethanol and 1,2-dimethoxyethane (Figure 5A) does not simply follow the dielectric permittivity of the solvent but is rather governed by explicit-solvent effects such as hydrogen bonding, making them an ideal test case. For both compounds, the conformational ensemble can be described based on the rotational state of the central torsion angle (Figure 5A). In refs 40 and 55, ^1^H NMR measurements were conducted that allowed the determination of a total scalar coupling *J*_tot_ of the α proton of the methoxy group, whose magnitude is directly related to the population of the conformations of the central torsion in different solvents given two commonly made assumptions: First, the experimental *J*_tot_ is a population average over two distinct conformers (i.e., the *gauche*- and *trans*-conformer), and second, the underlying *J*_HH_ coupling constants of a given conformer are solvent independent. The conformers of the two compounds were evaluated using a higher level of theory as the molecular balances with the B3LYP functional^56–59^ and def2-TZVP basis set.^51^ Again, the three different implicit-solvent models were used in the QM calculations to optimize the two compounds in the ten solvents for which *J*_tot_ value had been determined experimentally. The correlation between the predicted population of the *trans*-conformer and the experimentally observed *J*_tot_ is shown in Figure 5.

**Figure 5:**
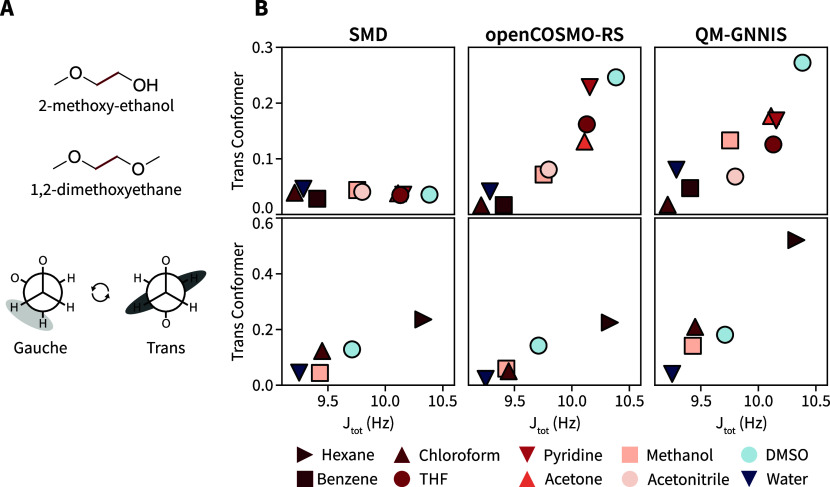
(A) Structure of 2-methoxy-ethanol and 1,2-dimethoxyethane and their shared Newman projection of the *gauche*- and *trans*-conformer. (B) Comparison of the predicted population of the *trans*-conformer and the experimental *J*_tot_ coupling constant of 2-methoxy-ethanol (top) and 1,2-dimethoxyethane (bottom) for QM calculations with SMD (left), openCOSMO-RS (middle), and QM-GNNIS (right). The color indicates the dielectric permittivity of the solvent (red: apolar, blue: polar).

For both compounds, the QM calculations with openCOSMO-RS and QM-GNNIS show a good correlation between their predicted populations and the experimental *J*_tot_ values. In contrast, QM calculations with SMD show no correlation for 2-methoxy-ethanol and a weaker one for compound 1,2-dimethoxyethane. This finding is especially interesting as the SMD implicit-solvent model showed the best correlations for the molecular balances (Figure 3), indicating that this good performance may likely be only because of the strong correlation with the dielectric permittivity of the solvent in case of the molecular balances. As alluded to in the Introduction (Figure 1), the relatively low population of the *trans*-conformer in water may be attributed to the formation of a pseudoseven-membered ring via a hydrogen-bond network of a water molecule with 2-methoxy-ethanol.^40^ The lower *trans*-conformer population in water predicted by QM-GNNIS and openCOSMO-RS with respect to other polar solvents (i.e., methanol and DMSO) demonstrates that the approach can capture more complex solute–solvent interactions.

#### Benchmarking of Functionals and Basis Sets

A major advantage of the proposed approach is that because no QM data is required for training, the QM-GNNIS model should by definition be compatible with any functional and basis set combination. To demonstrate that this is true in practice and to analyze the influence of different functionals and basis sets, we have chosen five functionals (BP86,^50,60^ TPSS^61^ B3LYP,^56–59^ M06-2X,^62^ and ω-B97X^63^ and four basis sets (def2-SVP,^51^ 6-311G,^64^ def2-TZVP,^51^ and ma-def2-TZVP^51,65^ ) and tested all combinations for 2-methoxy-ethanol in three solvents: chloroform (lowest *J*_tot_), methanol (middle *J*_tot_), and DMSO (highest *J*_tot_). The results are summarized in Figure 6.

**Figure 6:**
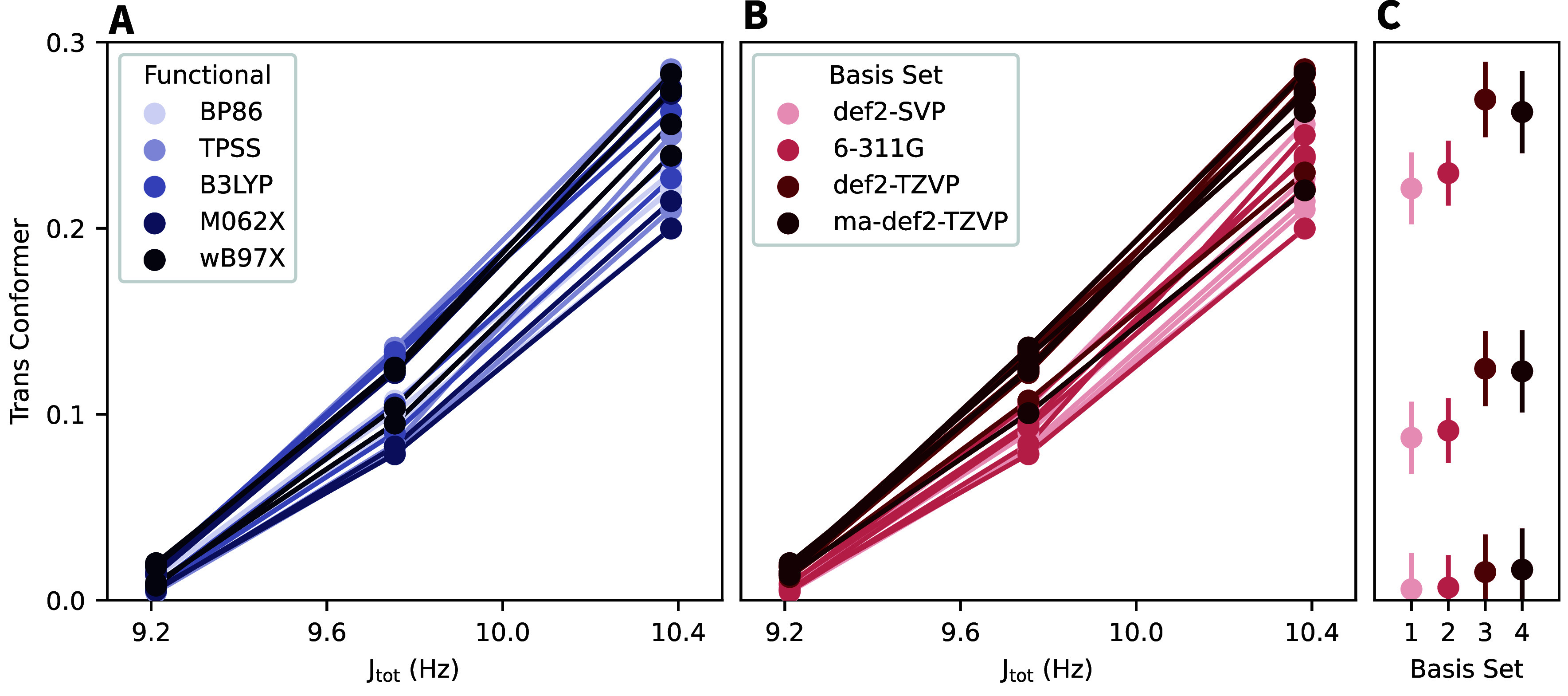
Comparison of the predicted population of the *trans*-conformer and the experimental *J*_tot_ coupling constant of 2-methoxy-ethanol for QM calculations with the QM-GNNIS model with different functionals and basis sets. In total, 20 combinations are shown for chloroform, methanol, and DMSO. For clarity, the same data is shown in three plots. (A) The color indicates all data points acquired with the same functional. (B) The color indicates all data points acquired with the same basis set. (C) Each point shows the average and standard deviation for all functionals used with the same basis set (1: def2-SVP, 2: 6-311G, 3: def2-TZVP, and 4: ma-def2-TZVP).

The data indicates that the approach is indeed compatible with all tested functional and basis set combinations, which is supported by the fact that the variation between different functionals using the same basis set is consistent. Further, all combinations reproduce the experimental trend well, which is encouraging. Interestingly, the larger basis sets appear to favor the *trans*-conformer more than the smaller basis sets. While it is not clear whether this behavior is more physical, we note that the *trans*-conformer is more favored in the QM calculations compared to the fully classical results in ref 40. Taken together, these findings suggest that a more accurate description of the physical interactions correlates with a stabilization of the *trans*-conformer.

#### Infrared (IR) Measurements

Solvent effects—especially by polar solvents—can also be observed in IR spectra. To the best of our knowledge, there is currently no implementation of analytical gradient or Hessian calculation for any COSMO-RS model, including openCOSMO-RS. It is our understanding that the derivation of a closed-form expression for gradients and higher-order derivatives for COSMO-RS is complicated by the presence of not continuously differentiable functions in the energy-interaction operator and the iterative solution procedure of the COSMO-RS equations. Therefore, (open)­COSMO-RS can only provide free-energy weights for frequencies obtained for structures optimized in the gas phase or with other implicit-solvent models like SMD. In contrast, the (QM-)­GNNIS model is fully differentiable and allows analytical evaluation of gradients as used for geometry optimization and Hessians, which is a requirement for predicting vibrational frequencies along with all other physical properties that can be expressed as a derivative of the energy. This feature is for instance used here for the prediction of the vibrational frequencies observed in IR measurements.

To further investigate the performance of the implicit-solvent models, the IR spectra of 2-methoxy-ethanol in five different solvents were predicted, and the OH vibrational frequencies were compared against experimental data from ref ^66^. Note that only the frequencies were predicted, which were analyzed using a Boltzmann weighting according to the predicted free energies of the conformers. The intensities resulting from a changed dipole derivative were assumed constant. The predicted OH vibrational spectra and the comparison of the frequencies against experiment are shown in Figure 7.

**Figure 7:**
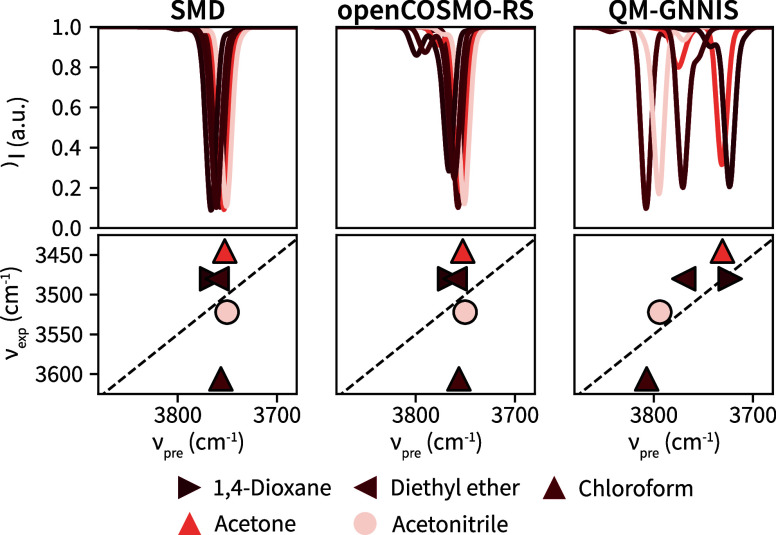
Predicted IR spectrum of the OH vibration based on QM calculations with SMD (upper left), openCOSMO-RS (upper middle), and QM-GNNIS (upper right). The comparison between the predicted (*x*-axis) and experimentally observed frequencies ν (*y*-axis) for each solvent is shown below the predicted spectra. The dashed black line indicates the line *y* = *x* + *k* where *k* is an offset.

While the predicted frequencies of the QM calculations with SMD and openCOSMO-RS are very similar to each other independent of the solvent, the calculations with QM-GNNIS show larger solvent effects, which is in agreement with experiment. Note that the absolute values of the computed versus experimental wavenumbers are shifted by approximately ∼200 cm^–1^ for all methods. We hypothesize that this shift is a result of the level of theory used, as it is well-known that frequencies computed with DFT methods show a constant offset.^67^

From Figure 7, we conclude that the QM-GNNIS model seems to capture key effects from the interaction between the solvent and the OH group of 2-methoxy-ethanol that give rise to a difference in the vibrational frequency unattainable by the other implicit-solvent models. This finding is especially striking as new conclusions can be drawn based on these results that provide an alternative explanation for the solvent-specific frequencies. In the original publication ref 66, the authors surmised that the difference in frequency resulted from an opening of the intramolecular hydrogen bond and concluded that the frequency shift was directly linked to the “open” vs “closed” pseudo five-membered intramolecular ring. Our results, however, indicate that it may rather be the subtle differences of the closed ring conformers in conjunction with the direct effect of the solvent interacting with the OH group that shift the vibrational frequency. A more detailed analysis indicates that (i) the closed-ring form dominates in all solvents, and that (ii) the opened-ring conformers feature higher rather than lower frequencies (see Figure S5), the opposite of what was suggested in ref ^66^. This observation highlights the proposed QM-GNNIS model as a versatile approach that can accurately predict the solvent-dependent conformational ensemble of molecules while also providing access to experimental properties such as IR frequencies.

## Conclusions

Here, we have presented a novel way of developing a machine-learned QM implicit-solvent approach. The method separates the task of modeling the solvent into evaluating a continuum-based QM implicit-solvent method and adding a machine-learned explicit-solvent correction term. This term can be parametrized from classical explicit-solvent MD simulations, which are known to capture solute–solvent effects well, thus requiring no additional QM/MM training data. The resulting QM-GNNIS model has been demonstrated to outperform state-of-the-art QM-based implicit-solvent methods and provides access to novel explanations for experiments. The gradients and Hessians, in addition to energies, enable broad applications, and the additive nature of the method allows easy implementation with existing QM software packages.

## Methods

For QM calculations, ORCA 6.0.1 ^68,69^ was used throughout this work. If not stated otherwise, ORCA’s default settings are applied. For COSMO-RS, the open-source implementation of COSMO-RS by Gerlach et al.^48^ was used and denoted as openCOSMO-RS. The ASE software^70^ was used to interface with ORCA and allow the integration of the QM-GNNIS model.

### Structure Minimizations

An overview of the proposed workflow for the different implicit-solvent models is provided in Figure 8. For all compounds, the starting structure (details given in subsections Molecular Balances and NMR Measurements below) was optimized with SMD^29^ using ORCA with the OPT keyword. The free energies of the optimized conformer were calculated with SMD using the Freq keyword. The free energies for openCOSMO-RS were calculated by adding the free energy of the given conformer in vacuum (obtained with the Freq keyword) to the solvation free energy calculated with openCOSMO-RS.

**Figure 8:**
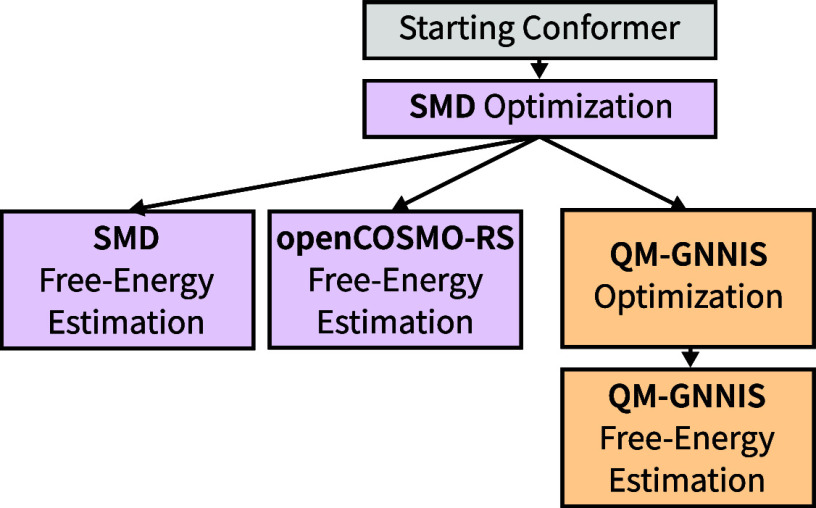
Schematic workflow of the different geometry minimizations and free-energy estimations performed in this study. The traditionally applied workflow using SMD and openCOSMO-RS is highlighted in purple. The approach with QM-GNNIS is shown in orange.

For GNNIS, the molecule-dependent parameters (i.e., atomic partial charges and van der Waals radii) of the GNN were set to the same values as for the classical GB-Neck2 model^35^ as described in ref 40 . The preoptimized structure was further optimized using the BFGS algorithm^71^ with a tolerance of 0.025 eV angstrom^–1^ as implemented in the ASE package by combining the forces obtained with GNNIS and a QM calculation with CPCM.^72^ The dielectric constant used in the CPCM method was set to the same experimental value as in the GNNIS model (see ref 40 for exact values). The free energy of the further optimized structure *G*(r⃗) was evaluated according to,

2 $$G\left(\mathrm{r⃗}\right)=E_{\mathrm{CPCM}}^{\mathrm{QM}}\left(\mathrm{r⃗}\right)+G_{\mathrm{GNNIS}}^{\mathrm{MM}}\left(\mathrm{r⃗}\right)-G_{\mathrm{GB‐Neck2}}^{\mathrm{MM}}\left(\mathrm{r⃗}\right)-TS_{\mathrm{CPCM‐GNNIS}}^{\mathrm{QMMM}}\left(\mathrm{r⃗}\right)$$

where $E_{\mathrm{CPCM}}^{\mathrm{QM}}\left(\mathrm{r⃗}\right)$ is the single-point QM energy with CPCM, $G_{\mathrm{GNNIS}}^{\mathrm{MM}}\left(\mathrm{r⃗}\right)$ is the free energy calculated with GNNIS, $G_{\mathrm{GB‐Neck2}}^{\mathrm{MM}}\left(\mathrm{r⃗}\right)$ is the free energy calculated with GB-Neck2, *r⃗* are the atomic coordinates, *T* is the temperature, and $S_{\mathrm{CPCM‐GNNIS}}^{\mathrm{QMMM}}$ is the vibrational entropy estimated using Grimme’s quasi-RRHO approach^73^ based on the combined Hessian of $\nabla^{2}E_{\mathrm{CPCM}}^{\mathrm{QM}}\left(\mathrm{r⃗}\right)+\nabla^{2}G_{\mathrm{GNNIS}}^{\mathrm{MM}}\left(\mathrm{r⃗}\right)-\nabla^{2}G_{\mathrm{GB‐Neck2}}^{\mathrm{MM}}\left(\mathrm{r⃗}\right)$ .

#### Molecular Balances

The structures of the studied molecular balances are shown in Figure 2 and the experimentally determined free-energy differences between the indicated ketone rotational states using ^19^F­{^1^H} NMR measurements were taken from ref 49. The starting structures for the molecular balances were taken from ref 40. There, a representative ensemble based on 5120 KDG^12^ conformers was generated using the minimization approach with the classical GNNIS model for each balance in each of the nine solvents. These structures were then optimized using the BP86 functional^50^ with the SVP^51^ basis set and a D4 correction.^52^ Next, their free energies were assessed in the nine different solvents for all implicit-solvent models according to the above description. Note that for QM-GNNIS, some conformers showed imaginary frequencies after the ASE minimization. As these do not represent true minima, these conformers were excluded from further analysis. The free energies of the minimized conformers were then used to calculate the populations of the two rotamers based on the ketone rotation shown in Figure 2. In order to focus the analysis on the differences in these free-energy differences, ΔΔ*G*, with respect to different solvents, the means of the experimental and predicted free energies were subtracted from the experimental and predicted free energies, respectively, for each balance. Note that data points, which only represent upper boundaries (i.e., populations lower than the detection limit of the NMR procedure), were not included in the comparison. SciPy^74^ (version 1.10.1) was used to calculate the Pearson correlation coefficient (PCC) and the linear least-squares regression between the experimental and predicted ΔΔ*G*. The comparison based on the regression slopes was performed on the log scale. Anticorrelated examples with negative slopes were assigned log values of minus infinity. This was the case for three balances (D2, D5, and C6) when using the openCOSMO-RS model. The significance of comparisons was assessed using the Wilcoxon signed-rank test. *p*-values below 0.05 were considered significant.

#### NMR Measurements

Experimentally obtained *J*_tot_ couplings of 2-methoxy-ethanol at 25 °C and 1,2-dimethoxyethane at 40 °C were taken from refs 40 and 55, respectively. For each compound, 255 starting structures were obtained with the KDG^12^ conformer generator and optimized for all solvent and implicit-solvent model combinations using the B3LYP functional^56–59^ with the TZVP basis set^51^ and a D4 correction.^52^ The minimized structures were subsequently sorted based on their ascending free energies, and the ensemble was pruned using MDTraj^75^ (version 1.9.7) based on the all-atom RMSD with a threshold of 0.05 nm. The resulting conformers in the ensemble were assigned to the *gauche*-conformer if the central dihedral angle (see Figure 5) was between −2.1 and 2.1 rad, and to the *trans*-conformer otherwise. The population of the *trans*-conformer was then calculated according to the assigned free energies.

#### Benchmarking of Functionals and Basis Sets

For the benchmarking of the functional and basis sets, the same procedure as described above was applied with five different functionals (BP86,^50,60^ TPSS^61^ B3LYP,^56–59^ M06-2X,^62^ and ω-B97X^63^ and four basis sets (def2-SVP,^51^ 6-311G,^64^ def2-TZVP,^51^ and ma-def2-TZVP^51,65^ ) for chloroform, methanol, and DMSO.

#### IR Measurements

Experimental measurements of the OH IR frequencies of 2-methoxy-ethanol were taken from ref 66. The same optimization process as used for the NMR comparison was applied. The vibrational frequencies in the QM calculations with SMD and QM-GNNIS were calculated based on the Hessians obtained during the free-energy calculations. The frequencies were broadened using Gaussians with a σ of 5 Hz, and the total spectrum was obtained as the population-weighted sum over all conformers. As openCOSMO-RS does not allow the calculation of Hessians, the frequencies of the SMD model were used with the population weights obtained using openCOSMO-RS.

## Supplementary Material



## Data Availability

The code used in this study is freely available on GitHub (https://github.com/rinikerlab/QM-GNNIS). The data set used to train the GNNIS model from ref 40 is freely available in the ETH Research Collection (DOI: 10.3929/ethz-b-000710355).

## Notes

Comparison of SMD versus CPCM and additional figures (PDF)
