# Supplementary material for: Transferring Knowledge from MM to QM: A Graph Neural Network-Based Implicit Solvent Model for Small Organic Molecules
Source: J Chem Theory Comput. 2025 Jul 28;21(15):7450–9. doi: 10.1021/acs.jctc.5c00728 (PMC12355693; doi:10.1021/acs.jctc.5c00728)
Supplement: Supplementary file 1 [file ct5c00728_si_001.pdf]

SUPPORTING INFORMATION
Transferring Knowledge from MM to QM: A Graph Neural Network Based Implicit Solvent Model for Small Organic Molecules

Paul Katzberger[‡], Felix Pultar[‡], and Sereina Riniker[*]

[*] *Department of Chemistry and Applied Biosciences, ETH Zurich, Vladimir-Prelog-Weg 2, 8093 Zurich, Switzerland. Email: sriniker@ethz.ch*

[‡] *Authors contributed equally to this work.*

Contents

S1 SMD versus CPCM **S2**

S2 Additional Figures **S3**

S1 SMD versus CPCM

To ensure that the choices made of combining the QM-GNNIS model with the CPCM solvent and comparing it to the SMD solvent are valid, we have performed the same pipeline as described in the main text in two variations: (i) we have evaluated the QM-GNNIS model with the SMD solvent (denoted as QM-GNNIS (SMD)), and (ii) we have carried out the initial minimization with the CPCM solvent and evaluated its performance. The results of these two additional approaches are shown in Figure S1.

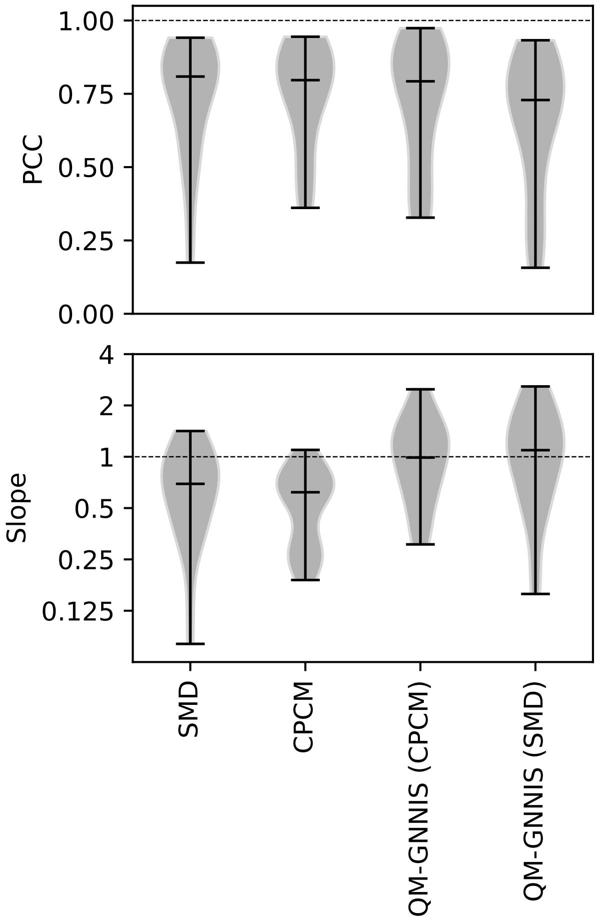

Figure S1: (**Top**): Pearson correlation coefficient (PCC) between the experimentally observed $\Delta\Delta G$ values and the predictions based on QM calculations with SMD, CPCM, QM-GNNIS (CPCM), and QM-GNNIS (SMD). (**Bottom**): Slopes between the experimentally observed $\Delta\Delta G$ values and the predictions based on QM calculations with SMD, CPCM, QM-GNNIS (CPCM), and QM-GNNIS (SMD).

While the Pearson correlation coefficients of SMD and CPCM are practically idential (0.81 vs. 0.80), the slopes with SMD are slightly closer to one than with CPCM (0.69 vs. 0.62). The latter indicate (slightly) stronger solvent-solute interactions for the SMD solvent than for the CPCM solvent, which could stem from the introduction of additional solvent-specific parameters in the SMD model. When combined with the QM-GNNIS model, the combination with CPCM does, however, outperform the SMD solvent with higher Pearson correlation coefficients (0.79 vs. 0.73) and slopes closer to one (0.99 vs. 1.1). These findings indicate that the QM-GNNIS model should be combined with the CPCM model and not the SMD model as the SMD model does already include some aspects about the (explicit) solvent that would lead to partial double counting when combined with the QM-GNNIS model.

S2 Additional Figures

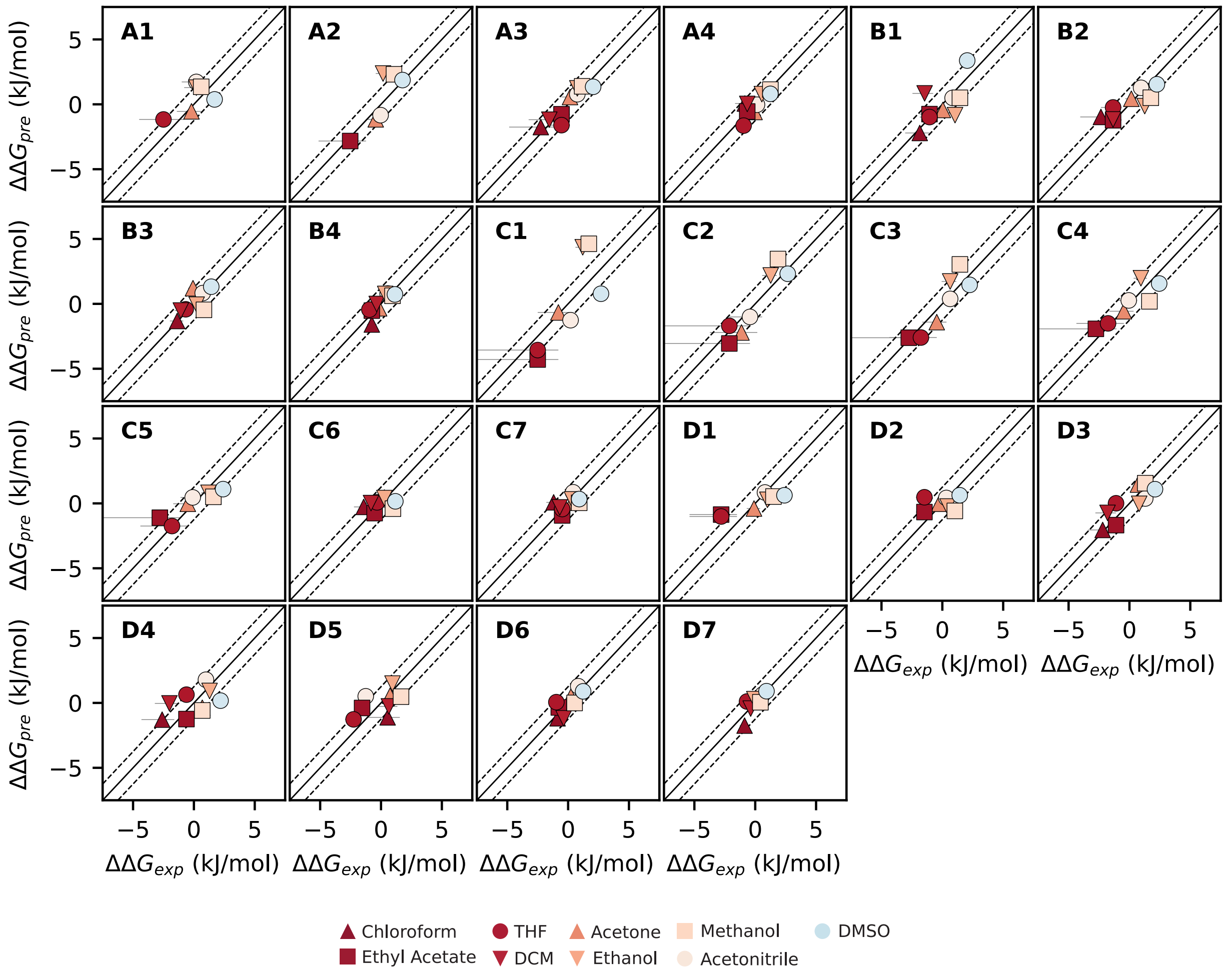

Figure S2: Comparison of the predicted versus observed free energy differences for the 22 molecular balances for the SMD model. The solid and dashed black lines indicate identity and deviations of half $\mathrm{k_b T}$, respectively.

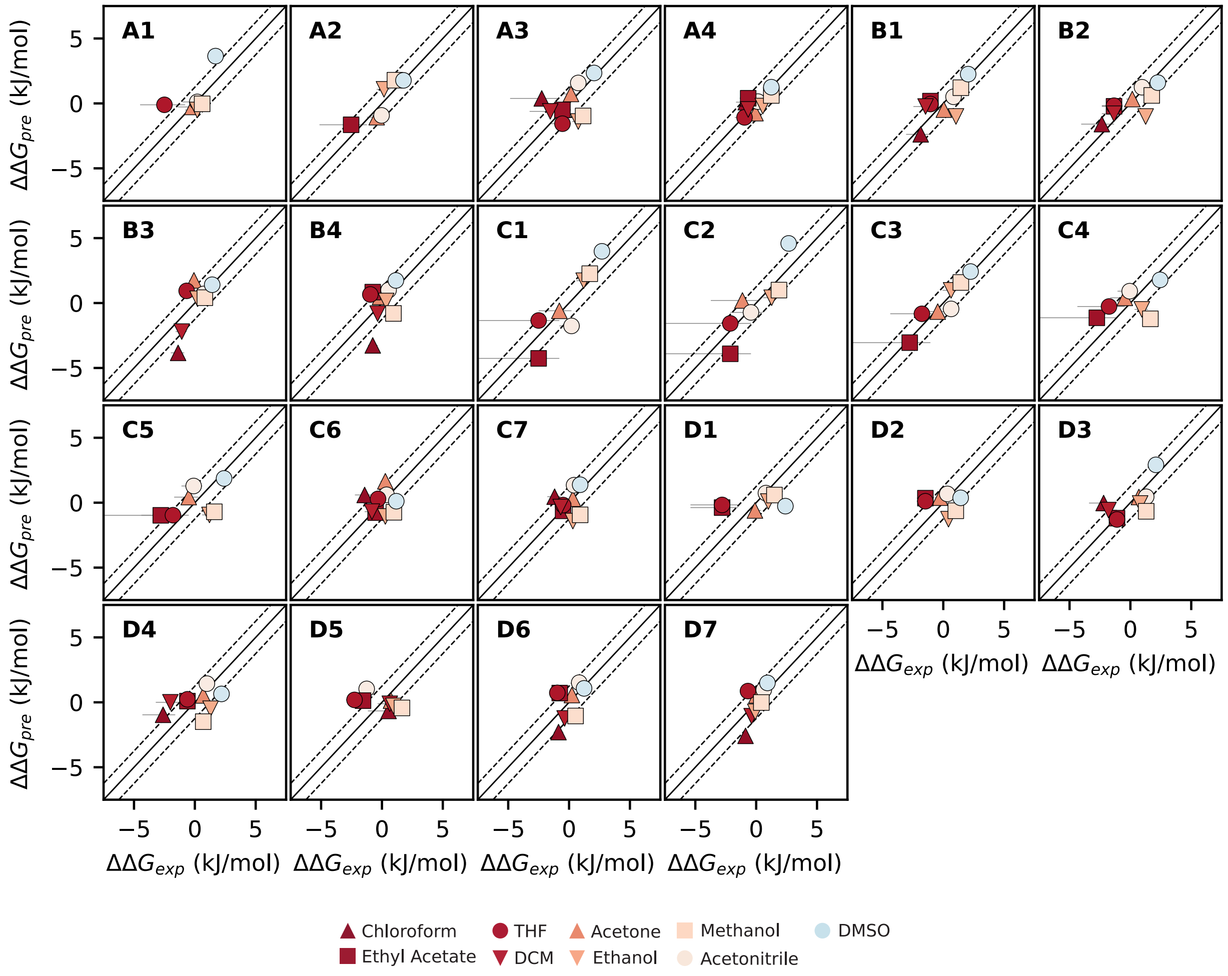

Figure S3: Comparison of the predicted versus observed free energy differences for the 22 molecular balances for the openCOSMO-RS model. The solid and dashed black lines indicate identity and deviations of half $\mathrm{k_b T}$, respectively.

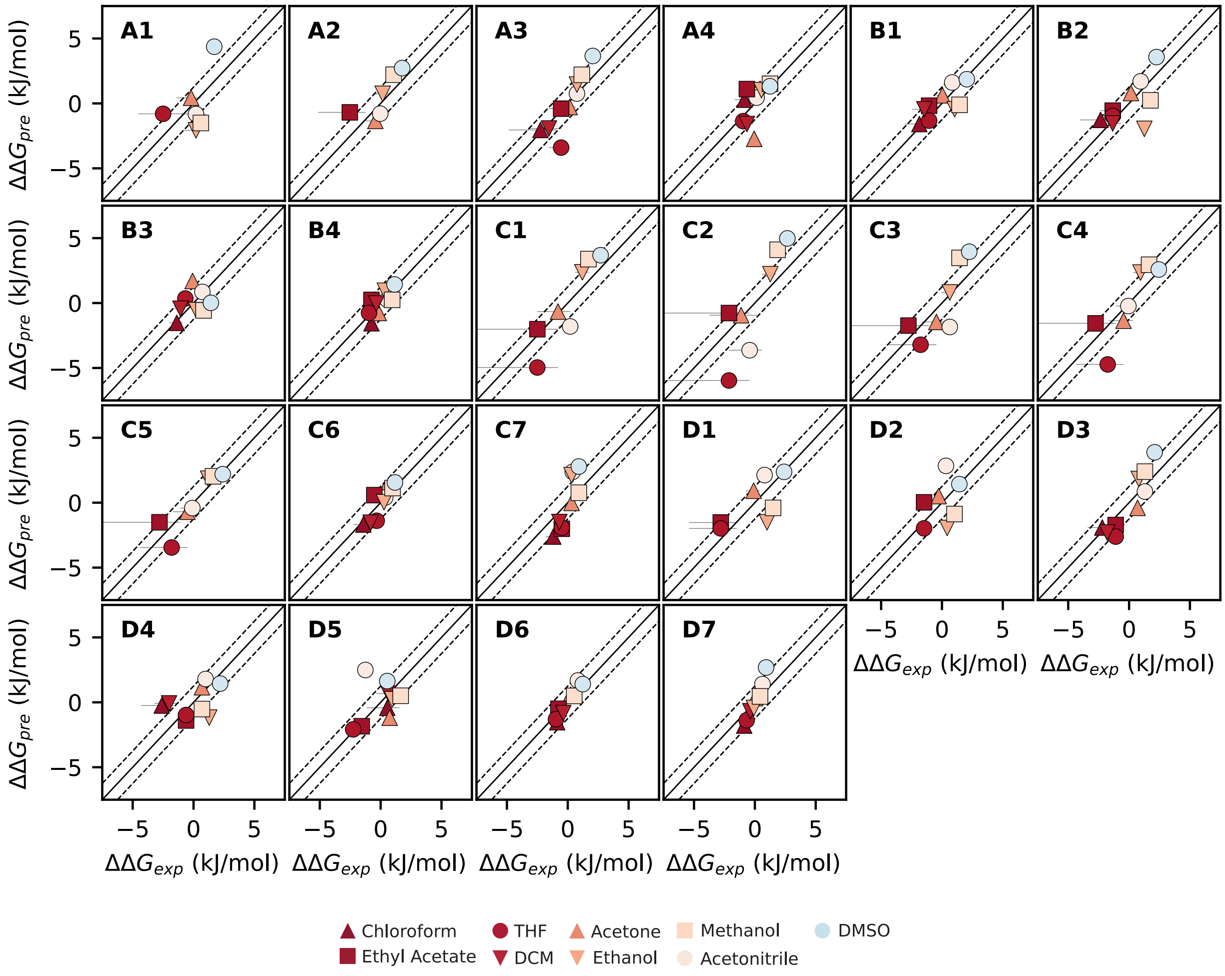

Figure S4: Comparison of the predicted versus observed free energy differences for the 22 molecular balances for the QM-GNNIS model. The solid and dashed black lines indicate identity and deviations of half $\mathrm{k_b T}$, respectively.

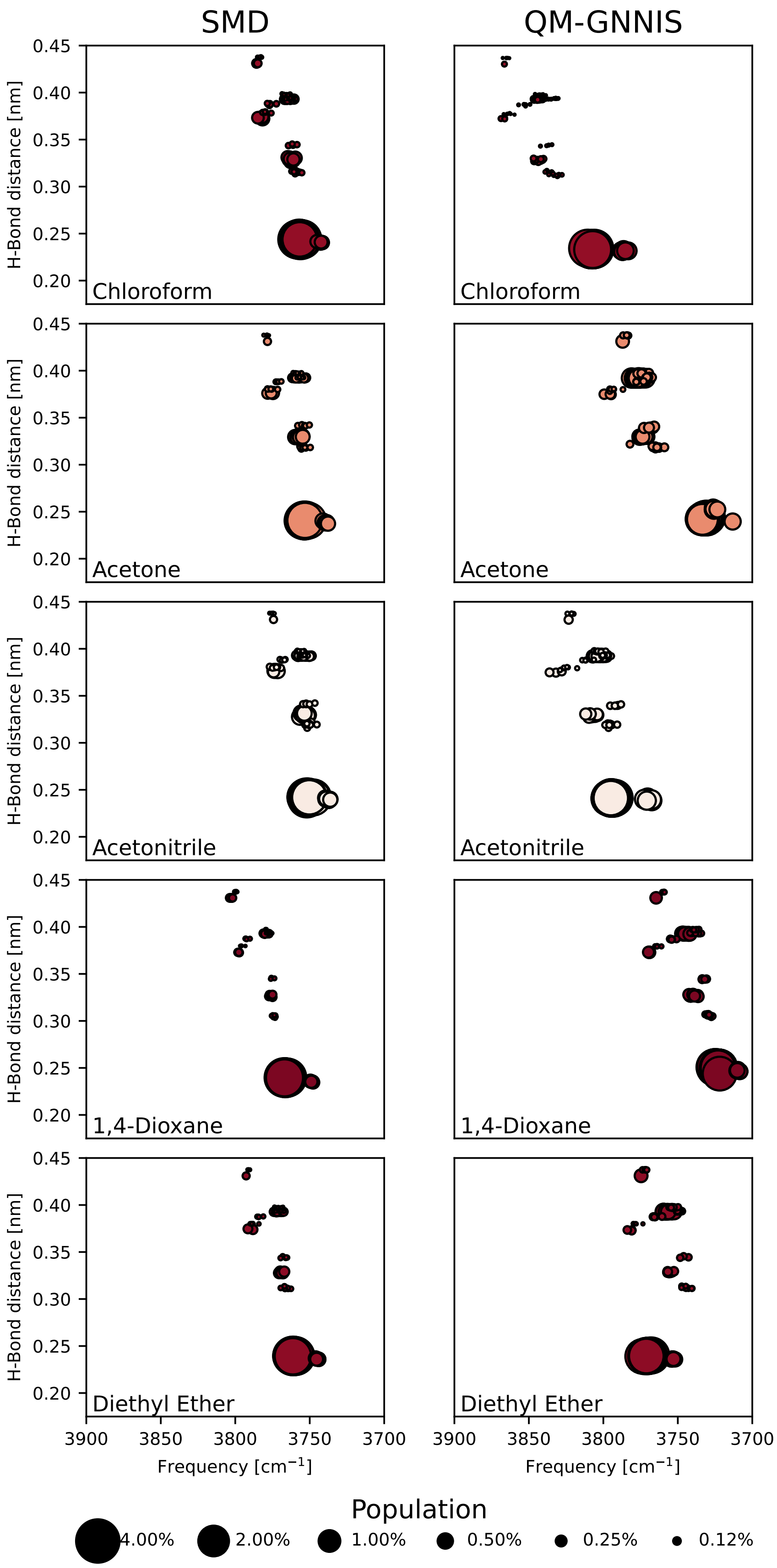

Figure S5: Comparison between the intramolecular hydrogen-bond distance and the predicted IR frequency for each conformer based on the SMD and QM-GNNIS model. All conformers featuring a hydrogen-bond distance of 0.275 nm were considered to be in the closed form. The marker size indicates the predicted populations.
